# Recanalization in Uncut Roux-en-Y Reconstruction: An Animal Experiment and a Clinical Study

**DOI:** 10.3389/fsurg.2021.644864

**Published:** 2021-08-06

**Authors:** Feng Wu, Zhizhan Ni, Hongliang Diao, Chenshen Huang, Song Wang, Bujun Ge, Qi Huang

**Affiliations:** ^1^Department of General Surgery, Tongji Hospital, School of Medicine, Tongji University, Shanghai, China; ^2^Department of General Surgery, Hwa Mei Hospital, University of Chinese Academy of Sciences, Zhejiang, China; ^3^Department of General Surgery, Karamay Central Hospital, Karamay, China

**Keywords:** gastric cancer, laparoscopic, uncut Roux-en-Y reconstruction, complication, distal gastrectomy

## Abstract

**Background:** Because of the challenge of jejunal closure recanalization, uncut Roux-en-Y reconstruction remains controversial. This study aimed to investigate the incidence of recanalization after uncut Roux-en-Y reconstruction in pigs and a small number of patients.

**Methods:** Twenty miniature pigs were subjected to distal gastrectomy and uncut Roux-en-Y reconstruction using various rows of linear staplers to block the intestine. The pigs were sacrificed, and the incidence of recanalization was investigated 1 month after the operation. From December 2018 to June 2019, 10 patients with gastric cancer who had undergone elective laparoscopy-assisted distal gastrectomy and uncut Roux-en-Y reconstruction were included in this study. The primary study outcome was recanalization of the afferent limb, demonstrated by gastrointestinal radiography 1, 3, and 6 months after surgery. Various numbers of staple lines across the afferent jejunal limb were applied for closure: 2 staple lines in 2 pigs, 4 staple lines in 6 pigs, 6 staple lines in 8 pigs, and 8 staple lines in 4 pigs.

**Results:** Complete recanalization was detected in all 20 pigs 1 month postoperatively. Recanalization was detected in five cases (50%) by gastrointestinal radiography. Among them, 1 case of recanalization was found in the 1st month after the operation, 2 cases were found in the 3rd month, and another 2 cases were found in the 6th month. Bile reflux was detected by endoscopy in 2 patients with recanalization.

**Conclusions:** The occurrence of afferent limb recanalization after uncut Roux-en-Y reconstruction is high, and using additional staplers alone cannot decrease the incidence of recanalization. Based on our study, uncut Roux-en-Y reconstruction is not recommended.

## Introduction

Gastric cancer is one of the most common malignancies, accounting for the second most cancer-related deaths worldwide (1). Distal gastrectomy with D2 lymphadenectomy followed by gastrointestinal tract reconstruction is recommended as a standard operation for patients with distal gastric cancer (2). Classical methods of gastrointestinal tract reconstruction include Billroth I, Billroth II, Roux-en-Y and uncut Roux-en-Y anastomosis (3). However, which anastomosis methods are superior remains controversial.

Compared with Billroth I and Billroth II reconstruction, the advantages of the Roux-en-Y method include reduced reflux gastritis and esophagitis (4, 5), a reduced incidence of surgical complications such as ruptured suture lines (6–8) and a decreased probability of gastric cancer recurrence (8–10). However, the procedure is much more complex than Billroth I and Billroth II reconstruction. Furthermore, unfortunately, symptoms associated with Roux stasis syndrome appear in ~30% of patients after Roux-en-Y reconstruction (11). Interruption of the continuity of the small intestine and altered duodenal electrical conduction cause symptoms, including upper abdominal distension, nausea, and vomiting (12, 13).

**Figure 1:**
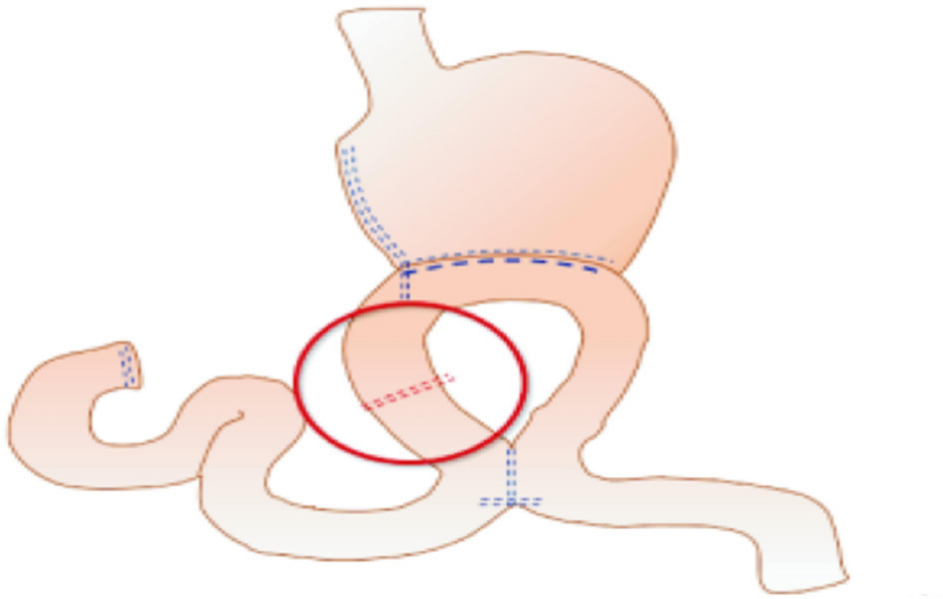
General view of the surgical procedures.

The uncut Roux-en-Y technique is an ideal method that addresses all problems theoretically. Van Stiegmann and Goff first reported uncut Roux-en-Y anastomosis in 1988 (14). It is a modification of the Billroth II reconstruction plus Braun reconstruction that blocks the afferent jejunum 3 cm proximal to the stump-jejunal anastomosis. The uncut Roux-en-Y method is shown in Figure 1. Because uncut Roux-en-Y reconstruction maintains jejunum continuity instead of cutting off the jejunum compared with the Roux-en-Y method, uncut Roux-en-Y reconstruction can remit Roux-en-Y stasis syndrome, preserve duodenal electrical continuity, and avoid the secondary emergence of ectopic pacemakers (15, 16). However, the incidence of recanalization remains a concern (17–21), reaching 2.9–35.7%, according to previous studies. Thus, further study is needed for this promising technique.

The present study aimed to compare the incidence of recanalization after uncut Roux-en-Y reconstruction using various rows of linear staplers to block the afferent limb in pigs. Additionally, the incidence of recanalization was evaluated in a small number of patients who were diagnosed with gastric cancer and had undergone laparoscopy-assisted distal gastrectomy followed by uncut Roux-en-Y reconstruction.

## Materials and Methods

### Animals

#### Experimental Pigs

The protocol was approved by the Shanghai Tongji Hospital Ethics Committee. Twenty miniature pigs (weight between 28 kg and 32 kg; 8 male and 12 female) had undergone distal gastrectomy and uncut Roux-en-Y reconstruction using various rows of linear staplers to block the intestine. The pigs were sacrificed 1 month after the operation, and recanalization was evaluated. The sex of the animal was not considered a factor that may affect the results.

#### Periprocedural Management

The animals were fasted overnight before the operation. Ketamine was used to sedate the experimental pigs at a dose of 22 to 30 mg/kg intramuscularly, followed by intravenous administration of 30 mg of propofol (10 mg/mL). After the pigs were anesthetized, a 12-cm upper midline incision was made. The distal stomach was carefully resected, and gastrointestinal tract reconstruction was then performed. For the uncut Roux-en-Y reconstruction, a side-to-side gastrojejunostomy was constructed 30 cm distal to the ligament of Treitz. Occlusion of the jejunal lumen was made 3 cm proximal to the anastomosis by various numbers of staple lines (TX60B No-Knife, double-row, 3.5-mm width; Ethicon PROXIMATE), and Braun's jejunojejunostomy was created 35 cm distal to the anastomosis.

All animals tolerated the procedure. Antibiotics and a liquid diet were administered on postoperative days (PODs) 1 and 2. Next, a normal diet was initiated on POD 3 depending on the general condition of the animals.

#### Evaluation of Recanalization

One month after the operation, the pigs were sacrificed, and recanalization was detected. At the time the animals were sacrificed, the whole abdominal cavity and site of the procedure were carefully examined for evidence of adhesion and disruption. Next, the reconstructed intestine was harvested. The stapled jejunum was assessed for gross recanalization by opening the jejunal loop above and below the partition site and checking the area for patency. Finally, the site of staple occlusion was cut open to assess the change in the mucosa.

### Patients

#### Ethics

Ethics approval for this study was reviewed and approved by Shanghai Tongji Hospital Ethics Committee (reference number 2018-LCYJ-005). Written consent was obtained from all the participants before surgery. The registration number of this study is ChiCTR-1800015228.

#### Participant Selection

From December 2018 to June 2019, 10 patients with gastric cancer from two hospitals [Shanghai Tongji Hospital (4 cases), Shanghai, China and Hwa Mei Hospital (6 cases), Ningbo, China] who had undergone elective laparoscopy-assisted distal gastrectomy (LADG) with D2 nodal dissection were included in this study. After dissection, uncut Roux-en-Y reconstruction was performed using a small upper abdominal midline incision. All surgeries were performed by two teams.

The patient inclusion criteria were as follows: (1) age ≥18 years; (2) clinical tumor stage I-III, evaluated as radically resectable; (3) documented informed consent; (4) ECOG score 0–1. The exclusion criteria were as follows: (1) other serious diseases, including cardiovascular, respiratory, kidney, and liver diseases, and poorly controlled hypertension or diabetes; (2) mental illness; (3) pregnant or lactating women; (4) preoperative neoadjuvant chemoradiotherapy; (5) emergency surgery; (6) history of gastric surgery; (7) forced expiratory volume in 1 s (FEV1) <50% FEV1 predicted.

#### Preoperative Management

Before surgery, gastroscopies, endoscopic biopsies, and computed tomography scans (CT) were performed to confirm the tumor size, location, and lymph node metastasis. Other examinations, such as electrocardiogram, echocardiographic, and lung function tests, were performed to evaluate cardiorespiratory function. The patients fasted for 6 h and were deprived of water for 2 h before the operation. After the induction of anesthesia, general anesthesia was performed through oral tracheal intubation. A nasogastric tube and a urinary tube were placed at the start of the surgical procedure.

#### Procedure

All patients had undergone LADG with D2 lymphadenectomy, which was performed as described previously (22, 23). After LADG and closure of the duodenal stump, side-to-side anastomosis was performed between the remnant stomach and jejunum, 30 cm from the ligament of Treitz. Next, side-to-side anastomosis between the jejunum, ~35 cm from the gastrojejunostomy, and the jejunum, ~5 cm from the ligament of Treitz, was performed. The afferent loop 3 cm from the gastrojejunostomy anastomosis was closed using three applications of a noncutting stapler (TX60B No-Knife, double-row, 3.5-mm width; Ethicon PROXIMATE) through a 5-cm midline incision that was extended above the observation port.

#### Postoperative Management

Enhanced recovery after surgery (ERAS) elements included the use of patient-controlled analgesia, early removal of urinary catheters, and early ambulation beginning on POD 1. We removed the nasogastric tube on POD 2 according to the recommendation (24). Patients were subsequently allowed to receive a clear-liquid diet. They were encouraged to consume a semisolid diet on POD 3 according to tolerance. The drain was removed when the aspirate was minimal, usually within 3 to 4 days.

#### Follow-Up

All patients were followed at stated intervals postoperatively for at least 6 months. Follow-up examinations included laboratory tests and radiography, CT, and endoscopy examinations. The primary study outcome was recanalization of the afferent limb, demonstrated by gastrointestinal radiography 1, 3, and 6 months after surgery. Reflux gastritis, esophagitis, bile regurgitation, and anastomotic ulcers were assessed by endoscopy 6 months after surgery. Adjuvant chemotherapy was initiated within 1 month postoperatively depending on the pathological results.

The baseline patient characteristics, including sex, age, comorbidities, operation duration and pathological TNM (pTNM) stage, were recorded. Postoperative data, including postoperative complications, the length of postoperative hospital stay, and the results of gastrointestinal radiography and endoscopy, were also collected.

### Statistical Analysis

The data are presented as means ± standard deviation for continuous variables and as numbers for categorical variables. For extremely skewed data, the variables were reported as medians (interquartile range).

## Results

### Animals

First, two staple lines across the afferent jejunal limb were applied in two pigs. Both pigs showed recanalization 1 month after the operation. Next, 4 staple lines were used to block the intestine in 6 pigs; however, the staple lines were disrupted completely in all of them. We tried 6 staple lines, representing the most powerful blocking method used clinically (25). Unfortunately, this approach failed to block the jejunum in 8 pigs. Finally, 8 staple lines were applied to confirm whether reduced recanalization with enhanced blockage was helpful. However, recanalization seemed to be inevitable in 4 pigs. In conclusion, complete recanalization was demonstrated in all 20 pigs 1 month postoperatively.

**Figure 2:**
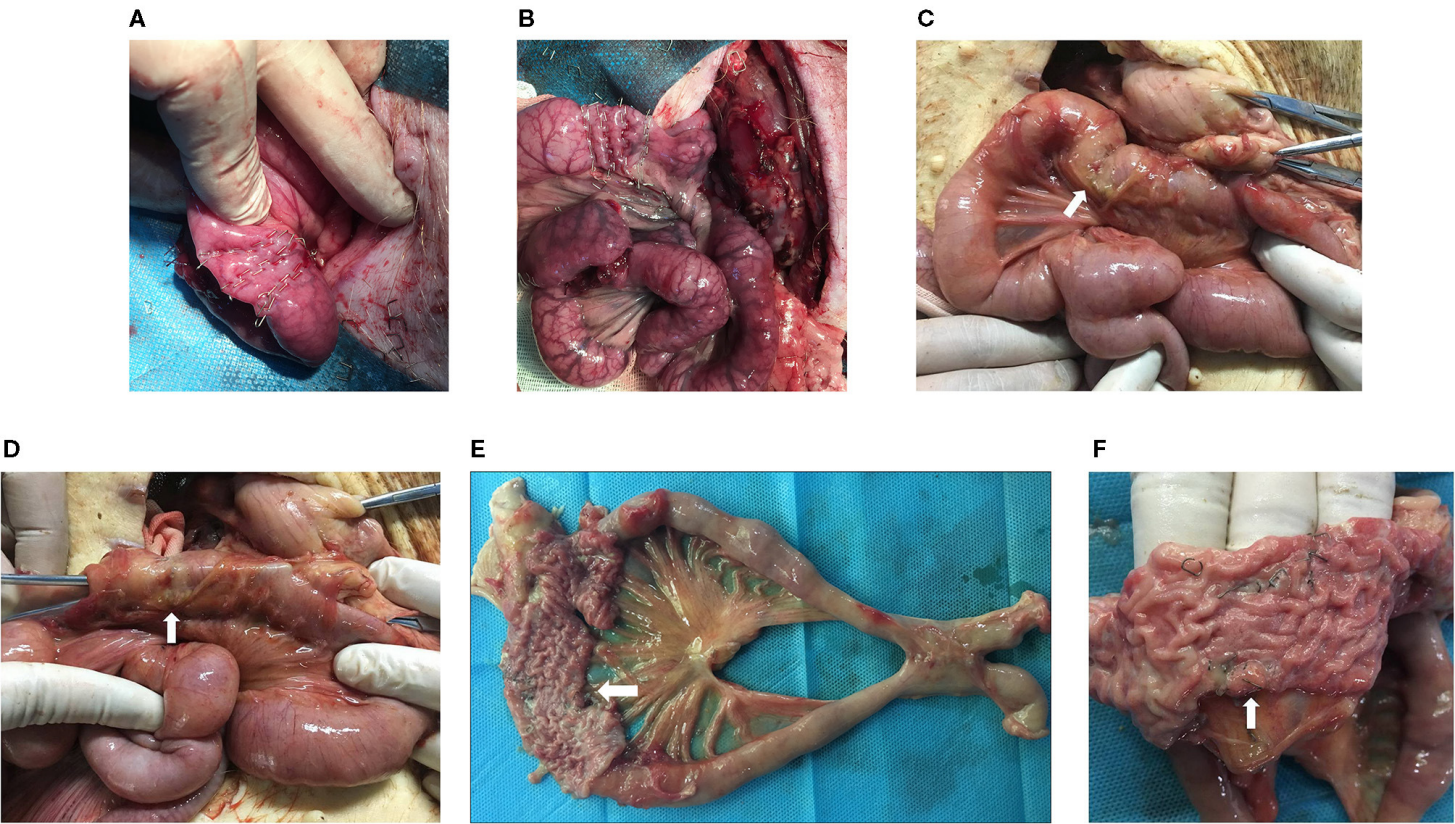
Animal experiments. The intestinal cavity on the afferent limb 3 cm from the gastrojejunostomy anastomosis was closed using 6-row linear staplers **(A)** and 8-row linear staplers **(B)**. After the pigs were sacrificed, the stapled site (white arrow) showed local indentation and a small amount of adhesion **(C)**. Recanalization was detected grossly by opening the jejunal loop above the stapled partition (white arrow) **(D)**. The operative site was harvested, and the stapled intestine (white arrow) was opened **(E)**. The residual staplers (white arrow) remained in the mucosa **(F)**.

During detection, the stapled site showed local indentation and a small amount of adhesion. No obvious leakage or staplers was observed on the serosal surface. After the stapled intestine was opened, residual staplers remained in the mucosa (Figure 2).

### Patients

#### Clinicopathological Characteristics

The demographic features of the 10 patients are shown in Table 1. The mean age was 63.6 ± 8.6 years. The main comorbidities were hypertension (5 cases), asthma (1 case), type B viral hepatitis (1 case), and type 2 diabetes mellitus (1 case). All laparoscopic operations were performed successfully without conversion to open surgery. The mean surgical duration was 192.4 ± 37.5 min. The mean length of postoperative hospital stay was 10.1 ± 2.2 days. The median follow-up duration was 11 months (range: 9–15). The postoperative pathologic stages based on the 8th edition of the American Joint Committee on Cancer (AJCC) cancer staging were as follows: 4 patients were stage I, 1 patient was stage II, and 5 patients were stage III. One patient developed pneumonia requiring antibiotics postoperatively. No perioperative mortality occurred in this series.

**Table 1:** Details of the 10 patients who had undergone uncut Roux-en-Y.

**Case no**.	**Sex**	**Age, y**	**pTNM**	**Operation duration, min**	**Recanalization time, m**	**Bile reflux**
1	M	68	T1bN0M0	195	3	No
2	M	72	T4aN2M0	137	–	No
3	M	72	T3N3M0	177	–	No
4	M	56	T3N0M0	202	6	Yes
5	M	73	T4aN2M0	231	3	No
6	M	70	T4aN2M0	169	–	No
7	M	52	T1aN0M0	228	–	No
8	M	61	T1aN0M0	160	–	No
9	M	48	T1bN1M0	265	1	Yes
10	M	64	T3N3M0	160	6	No

*M, male; pTNM, pathological TNM stage*.

#### Recanalization of the Afferent Limb

**Figure 3:**
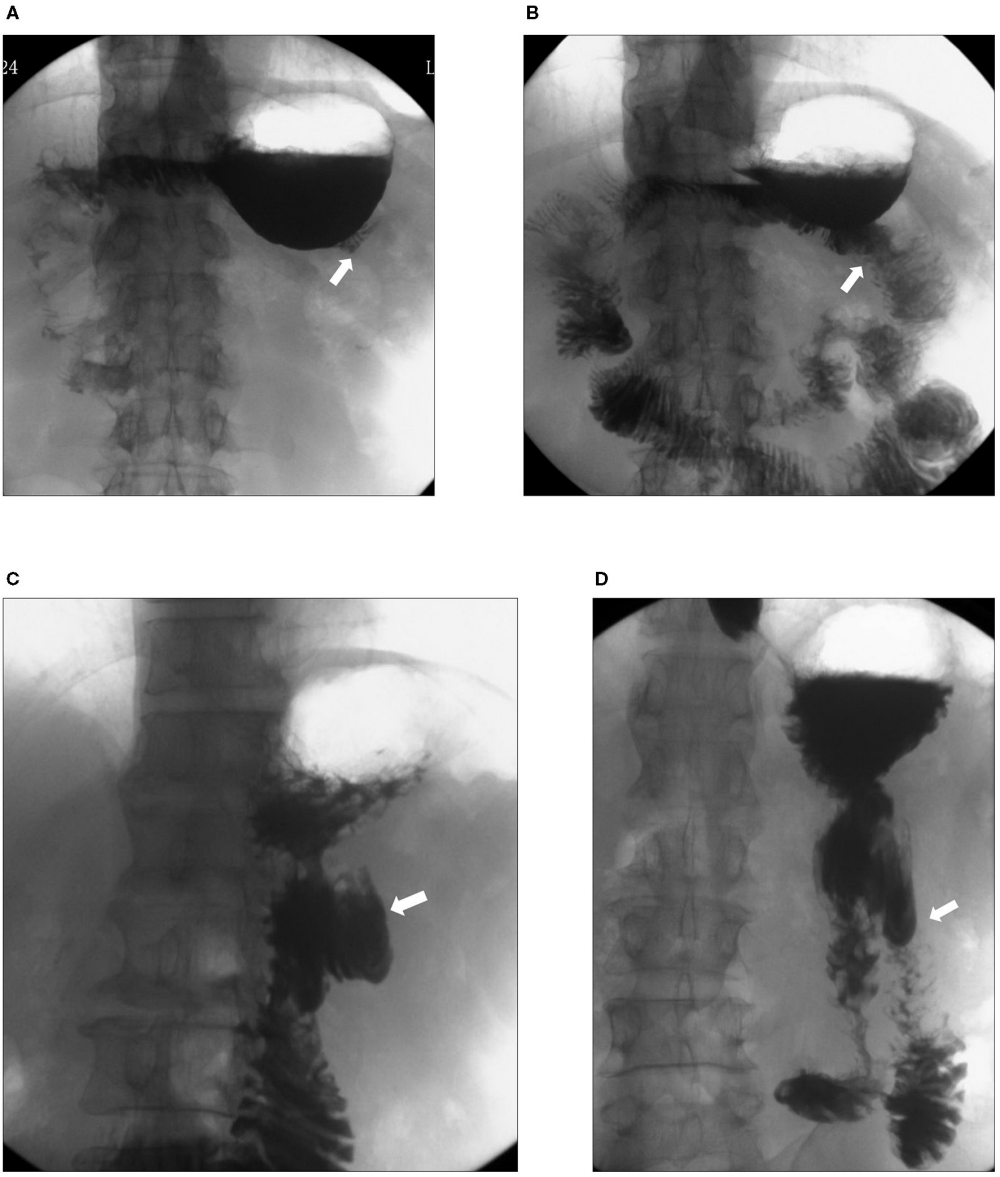
Upper gastrointestinal radiography of patients with recanalization. Upper gastrointestinal radiography showed closure of the afferent limb (white arrows) 1 month after the operation **(A)** and recanalization (white arrow) 3 months after the operation **(B)**. In another patient, upper gastrointestinal radiography showed closure of the afferent limb (white arrow) 3 months **(C)** after the operation and recanalization (white arrow) 6 months after the operation **(D)**.

**Figure 4:**
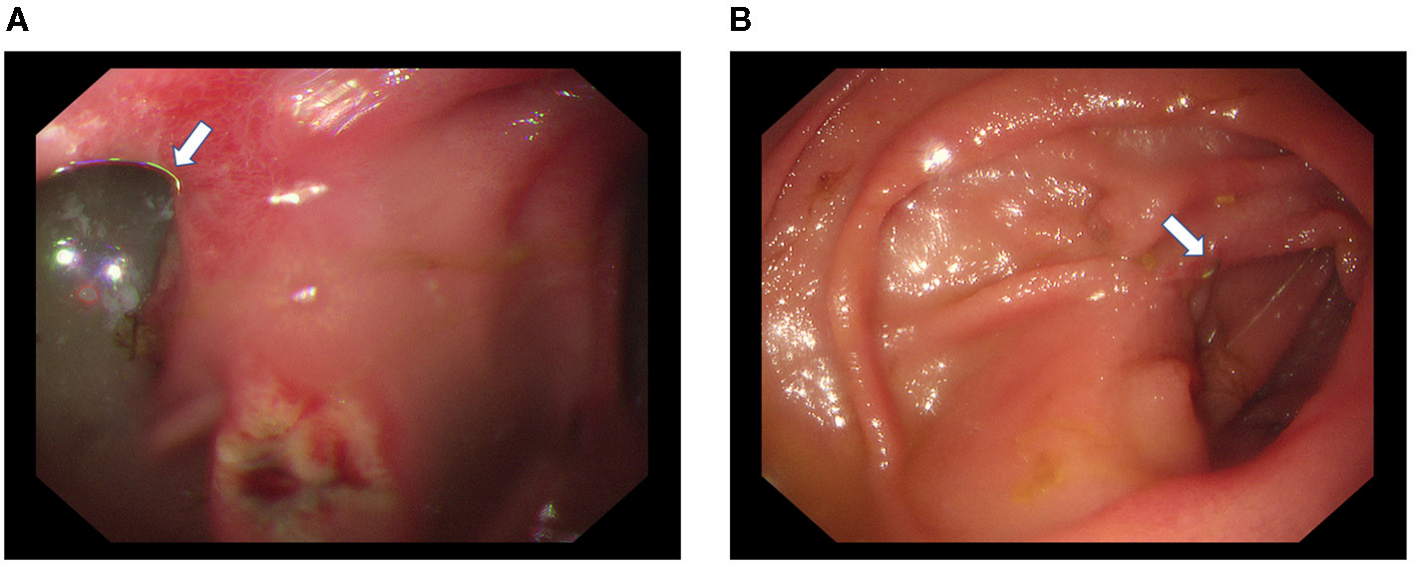
Gastroscopy of one patient with recanalization. Bile reflux (white arrow) through the afferent limb and a stomal ulcer were detected during endoscopy **(A)**. The residual staplers (white arrow) remained in the mucosa of the afferent limb **(B)**.

Recanalization was detected in five cases by gastrointestinal radiography (Figure 3). Among them, 1 case was found during the 1st month after the operation, 2 cases were found during the 3rd month, and another 2 cases were found during the 6th month. Bile reflux was detected by endoscopy in 2 patients with recanalization (Figure 4).

## Discussion

The benefit of uncut Roux-en-Y reconstruction to improve the oncological outcomes has long been debatable because of the high recanalization rate. Related studies have reported recanalization after uncut Roux-en-Y anastomosis, resulting in a higher incidence of alkaline reflux gastritis and esophagitis and affecting the quality of life (26, 27). In 1993, 5 patients (5/14) reported unsatisfactory results (mean follow-up of 296 days) after uncut Roux-en-Y reconstruction at the Mayo Clinic (17). Reoperation was performed in 4 patients, and recanalization because of disrupted staple lines was found in all 4 cases. A recent meta-analysis showed that the incidence of partial recanalization after uncut Roux-en-Y reconstruction reached from 2.9–13.0% after follow-up for 1 year (28). However, in this study, the recanalization rate soared to 100% 1 month after the operation in animals. Up to 50% of patients showed recanalization within 6 months postoperatively, which is dramatically higher than that in previous reports. Considering that very limited animal experiments have been reported until now (19, 20), 20 pigs may be the largest scale of animals used in the area of uncut Roux-en-Y reconstruction. Although the sample size of patients was relatively small, the results were reliable because the procedure was strictly based on clinical recommendations, and the follow-up was sufficiently detailed to reveal recanalization.

Why is the recanalization rate after uncut Roux-en-Y reconstruction so high? One of the assumptions is that the degree of compression to the mucosa of the jejunum is not sufficient to produce a mucosal wound, which would allow healing to occur (29). Furthermore, the anastomoses of the uncut afferent limb are mucosa-to-mucosa, while the submucosa forms the healing basis of intestinal anastomoses (30). The submucosa comprises mainly coarse, loosely interwoven, collagenous and elastic fibers. It maintains the GI tract with most of its tensile strength and is responsible for anchoring the sutures that hold anastomosed bowel ends together (30, 31). The reason for the failure of intestinal luminal occlusion by staple lines is the lack of permanent submucosal fibrous healing at the site of blockage. The staple height and the spaces between the single staples also require consideration to avoid recanalization. We only used 3.5-mm width stapler in our experiment, and the spaces are unfixed. However, the results are less satisfactory. Staplers with different height will be used and the spaces between the staples will be adjusted to observe the recanalization in future experiment.

Recanalization or dehiscence of uncut staple lines inevitably leads to bile reflux. In the present study, bile reflux was detected by endoscopy in 2 patients with recanalization. However, based on the small sample size and limited observation time, the influence of recanalization on bile reflux requires further confirmation with the inclusion of more patients.

Some researchers have tried alternatives to afferent jejunal occlusion to avoid recanalization from the staple line, including creating a bovine pericardium buttress (20), using a Teflon buttress (19), conceiving a new type of “uncut Roux” limb (32) and performing a luminal occlusion technique via a purse string suture with seromuscular reinforcement described by Noh (33). However, no method has been applied clinically with popular recognition until now. In the case of severe symptoms of bile reflux caused by recanalization, the best solution is to reoperate, through which conversion to a conventional Roux limb was accomplished (17).

Although pigs are widely used in surgical experiments, the differences between pigs and humans cannot be ignored. Particularly, the thinner intestinal wall, rougher food, and earlier resumption of a normal diet postoperatively in pigs may lead to different results. Additionally, because closure devices are specifically designed for humans, they may not be suitable for pigs. The higher rate and earlier onset of recanalization in pigs may be explained by all of the points mentioned above. The present clinical study is an exploratory study with a few cases; therefore, the exact recanalization rate will likely be clearer with a larger sample size. Additionally, further long-term observation is required to assess whether recanalization leads to symptoms of bile reflux and affects the patient's quality of life.

## Conclusion

Our study showed that the incidence of afferent limb recanalization after uncut Roux-en-Y reconstruction was high. Using more rows of staplers alone could not decrease the incidence of recanalization. Based on the results of the study, uncut Roux-en-Y reconstruction is not recommended. To improve the efficiency of blockage, new methods are required before the clinical application of uncut Roux-en-Y reconstruction.

## Data Availability Statement

The raw data supporting the conclusions of this article will be made available by the authors, without undue reservation.

## Ethics Statement

The studies involving human participants were reviewed and approved by Shanghai Tongji Hospital Ethics Committee (reference number 2018-LCYJ-005). The patients/participants provided their written informed consent to participate in this study. The animal study was reviewed and approved by Shanghai Tongji Hospital Ethics Committee. Written informed consent was obtained from the individuals for the publication of any potentially identifiable images or data included in this article.

## Author Contributions

QH was the main organizer of this study. ZN was responsible for writing the manuscript. FW, QH, and BG were the main surgeons. ZN, HD, and SW were the assistant surgeons. CH and FW performed the data analysis and completed the follow-up. All authors contributed to the article and approved the submitted version.

## Conflict of Interest

The authors declare that the research was conducted in the absence of any commercial or financial relationships that could be construed as a potential conflict of interest.

## Publisher's Note

All claims expressed in this article are solely those of the authors and do not necessarily represent those of their affiliated organizations, or those of the publisher, the editors and the reviewers. Any product that may be evaluated in this article, or claim that may be made by its manufacturer, is not guaranteed or endorsed by the publisher.
